# Avoidance behavior of juvenile common toads (*Bufo bufo*) in response to surface contamination by different pesticides

**DOI:** 10.1371/journal.pone.0242720

**Published:** 2020-11-30

**Authors:** Christoph Leeb, Sara Kolbenschlag, Aurelia Laubscher, Elena Adams, Carsten A. Brühl, Kathrin Theissinger

**Affiliations:** 1 iES Landau, Institute for Environmental Sciences, University of Koblenz-Landau, Landau, Rhineland-Palatinate, Germany; 2 LOEWE Centre for Translational Biodiversity Genomics, Senckenberg Research Institute, Frankfurt, Germany; University of Sassari, ITALY

## Abstract

Most agricultural soils are expected to be contaminated with agricultural chemicals. As the exposure to pesticides can have adverse effects on non-target organisms, avoiding contaminated areas would be advantageous on an individual level, but could lead to a chemical landscape fragmentation with disadvantages on the metapopulation level. We investigated the avoidance behavior of juvenile common toads *(Bufo bufo*) in response to seven pesticide formulations commonly used in German vineyards. We used test arenas filled with silica sand and oversprayed half of each with different pesticide formulations. We placed a toad in the middle of an arena, filmed its behavior over 24 hours, calculated the proportion of time a toad spent on the contaminated side and compared it to a random side choice. We found evidence for the avoidance of the folpet formulation Folpan® 500 SC, the metrafenone formulation Vivando® and the glyphosate formulation Taifun® forte at maximum recommended field rates for vine and a trend for avoidance of Wettable Sulphur Stulln (sulphur). No avoidance was observed when testing Folpan® 80 WDG (folpet), Funguran® progress (copper hydroxide), SpinTor^TM^ (spinosad), or 10% of the maximum field rate of any formulation tested. In the choice-tests in which we observed an avoidance, toads also showed higher activity on the contaminated side of the arena. As video analysis with tracking software is not always feasible, we further tested the effect of reducing the sampling interval for manual data analyses. We showed that one data point every 15 or 60 minutes results in a risk of overlooking a weak avoidance behavior, but still allows to verify the absence/presence of an avoidance for six out of seven formulations. Our findings are important for an upcoming pesticide risk assessment for amphibians and could be a template for future standardized tests.

## Introduction

About 40% of the area of the European Union is agriculturally used [1], making agriculture the dominant type of landscape in many regions. Modern agriculture is often linked to extensive use of agrochemicals to maximize crop yield. In 2017, 327 million kg of herbicides, insecticides and fungicides were sold in the EU to control pests, weeds, and diseases in agricultural fields [2]. This results in a contamination of most agricultural topsoils with pesticides [3, 4]. As breeding ponds of European amphibians can often be found within or near crops [5–7], amphibians are likely to come in contact with pesticides and contaminated soils during their pre- or post-breeding migration [8–10] possibly resulting in an uptake of pesticides [11, 12]. As the exposure to pesticides can have sublethal [13–15] and even lethal [16–18] effects, physiological and behavioral adaptations of amphibians to pesticides would decrease the hazard. Indeed, several studies found evidence for evolved pesticide tolerance in terms of decreased sensitivity in amphibian larvae of populations frequently exposed to pesticides, e.g. in *Lithobates sylvaticus* [19] or *Rana temporaria* [20]. The simplest behavioral response to minimize adverse effects might be to avoid a contamination. Such a response presupposes that amphibians are able to sense it.

Amphibians have good olfactory perception [21, 22], and a pesticide-permeable skin [23] allowing the uptake of large molecules [24]. Additionally, some pesticides used in agriculture are considered to be skin-irritating for humans, which is most likely also true for amphibians. Therefore, amphibians might be able to perceive contaminations and to assess the quality and suitability of water and surfaces to avoid them [25]. Several mesocosm and laboratory experiments investigated the avoidance of contaminated water bodies [26–28] as well as surfaces like soil or filter paper [11, 29–32]. Results are partly contradictory and might depend on the species, the substrate, the exposure period, the contaminant, and its concentration. Field studies that support surface laboratory tests are scarce, but some showed that amphibians tend to avoid arable fields as habitat and prefer non-cultivated areas [8, 33, 34]. Also genetic studies suggested a barrier effect of agricultural fields [35, 36]. However, it remains unknown if these effects are partly caused by pesticides or if they are solely the results of habitat characteristics.

For European amphibian species, studies on the avoidance of contaminated surfaces are lacking. Therefore, in the present study, we investigated the avoidance behavior of the common toad (*Bufo bufo* Linnaeus, 1758) in response to surface contamination by seven different pesticide formulations. We performed a laboratory experiment in which juvenile toads could choose between a contaminated and an uncontaminated side of a test arena. In general, our setup is comparable with those used in previous studies [11, 29–31], but instead of determining the side choice in intervals of minutes to hours, we continuously filmed the behavior of a toad in the arena over 24 h. Based on this video material, we answered the question if *B*. *bufo* avoids surfaces that had been contaminated with pesticides at 100% and 10% of the maximum recommended field rate. Continuous filming requires specialized hardware and, as it results in hundreds of hours of videos, also specialized tracking software to analyze the data. This comes with limitations in the experimental setup, e.g. the contrast between the surface and the experimental animal has to be high enough to allow a reliable tracking. Therefore, we tested if a reduced data set, which would also allow a manual analysis, results in the same pattern of potential avoidance behavior. As alterations of the movement behavior after pesticide exposure are well known for amphibian larvae [37], we further tested if the toads exhibit a different activity on the contaminated side of the arena.

## Material and methods

### Study species, sampling and animal husbandry

The common toad (*Bufo bufo* Linnaeus, 1758) is one of the most widespread amphibian species in Europe [38] and can be found in ponds within or near vineyards [6]. *Bufo bufo* is listed as “least concern” by the IUCN [39], but there are local declines of populations in their entire distribution area [40–43]. Although there is a trend to avoid vineyards as habitat, adult toads can be found directly in vineyards during their post-breeding migration and their risk for coming in contact with contaminated soil is high [8]. To investigate the potential of avoiding contaminated soils, we used juvenile toads because they are leaving their aquatic habitat between May and August in Germany [44], a time when most pesticides are applied in vineyards [8]. Further, juveniles play an important role in the dispersal and the population connectivity in many amphibian species [45]. Thus, an avoidance behavior of juveniles might have particularly adverse effects on the connectivity of populations.

Between the end of July and mid-September 2018 (see S1 Table for exact dates), juveniles of *B*. *bufo* (about 10 to 20 mm; metamorphed in June) were caught next to a permanent rainwater retention pond near Siebeldingen (Rhineland-Palatinate, Germany; 49.218368 N, 8.049538 E (WSG84); 196 m asl; S1 Fig). As the pond is used by hundreds of breeding individuals each year, we expect that the juveniles are from several different clutches. The pond is surrounded by a vegetative buffer strip, but is located in a landscape dominated by vineyards. As viticulture is a pesticide intensive crop with on average 9.5 pesticide applications per year in Germany [8, 46], the pond and the soils in the nearby vineyards can be expected to be contaminated with various agrochemicals. Thus, also toads using this pond can be expected to be regularly exposed to pesticides, both during their aquatic and terrestrial life stages. Collected toads were kept in groups of up to 40 individuals in outdoor net cages (40 x 65 x 30 cm) between six and 15 days (mean = 9.8 ± 4.3 days; see S1 Table for exact time spans) before an experiment. Individuals for the last choice-test (Wettable Sulphur Stulln) were only kept for one day. Cages were equipped with about 5 cm soil, moss and leaves as hiding places and were regularly watered with untreated tap water. Soil, moss and leaves were collected in the Palatinate Forest in a distance of about 1.6 km to the nearest vineyard and were therefore expected to be not contaminated with pesticides (S1 Fig). Toads were fed *ad libitum* with *Drosophila sp*. (own breed or purchased in a pet shop) or small insects ("meadow plankton") caught on a meadow where no pesticides are used (distance to the nearest vineyard = 2 km; S1 Fig). The day before an experimental run, animals were weighed to the nearest mg (CP153; Sartorius AG, Göttingen, Germany; see S1 Table for the mean weight of the individuals per experimental run), transferred into plastic boxes (11.5 x 17.5 x 13 cm) filled with about 2 cm of moist soil, moss and leaves and kept individually in the laboratory until the experiment. During this time the toads were not fed. Individuals chosen for an experimental run had been kept in the outdoor cages over the same time period. Further, we aimed to minimize the variance of the body weight within an experimental run. As common toads are explosive breeders we expected all individuals to have a similar age.

### Ethics statement

The study was approved by the Landesuntersuchungsamt in Koblenz (Germany; approval number G17-20-044). The collection of toads was permitted by the”Struktur- und Genehmigungsdirektion Süd Referat 42—Obere Naturschutzbehörde” (Neustadt an der Weinstraße, Germany; approval number 42/553-254/ 457-18(1)).

### Test substances

Experiments were performed with one insecticide, one herbicide and five different fungicide formulations (Table 1) that are frequently used in German vineyards and also in the area around the pond where toads were captured [8]. Commercial pesticides were obtained from a local distributor and the Julius Kühn-Institut (Siebeldingen, Germany). Three of the pesticide formulations are also approved for organic farming (Table 1). For each pesticide, the maximum recommended field rate (FR_max_) for vine was used. For four pesticides the test was also conducted with 10% of FR_max_. As we were limited in the number of performed test runs and most vineyards are managed conventionally, we tested only the conventional pesticides (Folpan® 500 SC, Folpan® 80 WDG, Taifun® forte, and Vivando®) with 10% of FR_max_. All stock solutions were prepared with tap water according to the manner of a common user for a water application rate of 200 L/ha.

**Table 1:** Pesticide formulations used for choice-tests with their maximum recommended field rate (FR_max_) for vine and the contained amount of active ingredient (A.I.).

Formulation	Type	A.I.	FR_max_ formulation	FR_max_ A.I.	Organic farming	CLP-Classification ^1^
Folpan® 500 SC ^2^	Fungicide	Folpet	2.4 L/ha	1.2 kg/ha	No	H315, H317
Folpan® 80 WDG ^2^	Fungicide	Folpet	1.6 kg/ha	1.28 kg/ha	No	H317
Funguran® progress ^3^	Fungicide	Copper hydroxide	2 kg/ha	1.074 kg/ha	Yes	-
SpinTor^TM^ ^4^	Insecticide	Spinosad	160 mL/ha	76.8 g/ha	Yes	-
Taifun® forte ^2^	Herbicide	Glyphosate	5 L/ha	1.8 kg/ha	No	H314
Vivando® ^5^	Fungicide	Metrafenone	320 mL/ha	160 g/ha	No	H317, H315
Wettable Sulphur Stulln ^6^	Fungicide	Sulphur	3.2 kg/ha	2.55 kg/ha	Yes	H315

The formulations were classified according to the Regulation (EC) No. 1272/2008 [CLP] [47].

^1^ At least the A.I. or one of the additives is classified according to Regulation (EC) No. 1272/2008 [CLP] as "Causes severe skin burns and eye damage" (H314), "Causes skin irritation" (H315), "May cause an allergic skin reaction" (H317). Other classifications that are not related to the skin were not considered.

^2^ ADAMA Deutschland GmbH; Cologne, Germany

^3^ Spiess-Urania Chemicals GmbH; Hamburg, Germany

^4^ DowDuPont Inc.; Wilmington, USA

^5^ BASF SE; Ludwigshafen am Rhein, Germany

^6^ Agrostulln GmbH; Stulln, Germany

### Experimental setup

All experiments were performed in glass petri dishes with a diameter of 20 cm filled with 300 g silica sand (SILIGRAN® dry, grain size: 0.1–0.3 mm; Euroquarz GmbH, Dorsten, Germany). We chose a bright sand to enhance the visual contrast of toads and background for subsequent filming. Prior to pesticide application, the sand was moistened with 29.85 mL of tap water (equivalent to 9,500 L/ha) by using a laboratory spray application system (Schachtner, Ludwigsburg, Germany). One half of each test arena was covered with a laminated paper semicircle (S2 Fig), and the pesticide stock solution was applied with the application system and an application rate of 200 L/ha. This resulted in a split design, with exactly one half of each test arena uncontaminated and one half contaminated with 0.31 ml of the pesticide solution. As the amount of pesticide is only about 2% of the amount of applied water, we neglected the resulting differences in the moisture between the two sides and did not apply additional water on the uncontaminated side. The test arena walls were then shielded with white paper strips to minimize external cues for the toads. To prevent escaping but still allow gas exchange and filming of the toads, each arena was covered with a polyamide fabric (sheer tights with 8 denier).

For one experimental run (i.e. one pesticide at one concentration; S1 Table) 16 replicates (i.e. 16 test arenas with one toad each; resulting in a total of 192 toads over the whole study) were used. Two arenas were placed in one dark test chamber (S3 Fig). The contaminated side of the arena was orientated randomly into one of the cardinal directions. An LED light was attached above each arena for illumination without shading the arena. A camera system, consisting of a Raspberry Pi (Raspberry Pi 3 Model B; Raspberry Pi Foundation, Cambridge, UK) with a camera module (SC15; Kuman Ltd., Shenzhen, China; S4 Fig) was attached to each test chamber. The camera was facing upside down to allow the filming of two arenas at the same time (S5 Fig). Videos were taken with a resolution of 1,296 x 730 pixels and 24 frames per second and saved as 30 or 60 min long H.264 files.

At latest 90 min after the application of the pesticides, one toad was placed in the center of a test arena and filming started for 24 h. The light was automatically turned off at 10 pm (about 10 h after test initiation) for 8 h. During this time, the arenas were illuminated with IR-light, which cannot be sensed by *B*. *bufo*, but allows continuous filming. Neither the test chambers nor the room with the test chambers had a sound insulation, but the room was not entered during any experimental run. Temperature during filming was 23 ± 2°C and the humidity between 57 and 81%. The toads were not fed during the time of the experimental run and were released in a distance of 200 m to the pond after the run.

Before the choice-tests with seven different pesticides, we conducted one control-test in completely uncontaminated arenas (n = 16) to exclude the presence of any external influences on the side choice or a preference for a cardinal direction.

### Video analysis

The recorded videos of the choice-tests were converted into MP4 files with the software XMedia Recode (Version 3.4.5.0; Sebastian Dörfler, Günthersleben-Wechmar, Germany). The software EthoVision^®^ XT (Version 12.0; Noldus Information Technology, Wageningen, Netherlands) was used to track the toads in the arenas. Toads were extracted from the background via dynamic and static subtraction. EthoVision^®^ XT determined every 0.4167 seconds (= sampling interval) if a toad was sitting in predefined zones within the arena (matching the contaminated and the uncontaminated side). Positions within a 2.5 cm wide area at the border between both sides (buffer zone) were excluded to take possible inaccuracies and unintended contaminations during the application process or leakage of the pesticide into account (see S1 Table for the mean time in the buffer zone per experimental run). Additionally, the distance moved between two time points was calculated. To reduce noise in the acquired tracks, track smoothing with a 2 mm threshold was used (method "minimal distance moved" with "direct" option in EthoVision^®^ XT). Tracks were checked for errors and reanalyzed with adjusted settings when necessary. Videos of the control-test were analyzed in the same way, but each arena was divided into halves orientated to the north & south and to the east & west.

### Parameters evaluated and statistical analysis

For statistical analysis, raw data from EthoVision^®^ XT were exported to R, version 3.4.3 [48]. To allow an acclimatization of the toads in the arenas, video material from the first three minutes of an experimental run were skipped during the analysis in EthoVision^®^ XT. Data from the following 12 minutes were excluded during the data analysis in R, resulting in a total acclimatization period of 15 minutes. For choice-tests, the percentage of time (*t*) an individual spent on the contaminated side of an arena (*t*_*pest*_) was calculated. To analyze if a reduction of the sampling interval affects the probability to detect an avoidance behavior, we subsampled the 24 hours of raw data and recalculated *t*_*pest*_ based on a sampling interval of 10 seconds (*t*_*pest_10*_), 60 seconds (*t*_*pest_60*_), 15 minutes (900 seconds, *t*_*pest_900*_) and 60 minutes (3,600 seconds, *t*_*pest_3600*_), starting with the first data point after the acclimatization period. Additionally, we reduced our data to the first hour of a choice-test (*t*_*pest_1h*_) without changing the sampling interval and thus ignored the remaining 23 hours of an experimental run. For the control-test, *t* was calculated for the side orientated to the north (*t*_*north*_) and west (*t*_*west*_). To identify a possible bias caused by the position of the arena within a test chamber or of the test chamber within the room, we calculated *t* also for the side of the arena orientated to the wall of the room (*t*_*wall*_) and to the second arena in the chamber (*t*_*arena*_). Both the direction to the wall and to the second arena correspond to a cardinal direction. Following the approach of Hatch et al. [29] and Gertzog et al. [31], two-sided one-sample Wilcoxon signed-rank tests were used to compare *t* to a theoretical value of 50% that can be expected from a random side choice for each experimental run. Additionally, two-sided paired Wilcoxon signed-rank tests were used to test for differences between *t*_*pest*_ and *t*_*pest_10*_, *t*_*pest_60*_, *t*_*pest_*900_, *t*_*pest_3600*_ or *t*_*pest_1h*_.

As additional behavioral endpoint for the choice-tests, the total distance moved per side (*d*) was calculated as measure of toad activity. To enable a comparison between moved distances on contaminated (*d*_*pest*_) and uncontaminated (*d*_*clean*_) sides, distances were corrected for the respective time spent per side and are given in meters per hour. As distances were not normally distributed, we used two-sided paired Wilcoxon signed-rank tests to test for differences between *d*_*pest*_ and *d*_*clean*_.

For all statistical tests, the criterion for significance was 0.05. When testing *t*_*pest*_ against 50% or *d*_*pest*_ against *d*_*clean*_, p-values from all tested formulations with the same concentration (n = 7 for 100% of FR_max_, n = 4 for 10% of FR_max_) were adjusted (p adj.) using the false discovery rate (FDR) method described by Benjamini and Hochberg [49]. As we wanted to see if the subsampling of the data would lead to the same avoidance pattern in a screening of the seven tested pesticide formulations, we also used FDR to adjust the p-values when testing *t*_*pest*_ against *t*_*pest_10*_, *t*_*pest_60*_, *t*_*pest_*900_, *t*_*pest_3600*_ and *t*_*pest_1h*_ in the same way. However, as we were also interested if the subsampling results in differences independent of the number of tested formulations in the screening, we also presented unadjusted p-values. P-values of the control-test and when testing *t*_*pest*_ against *t*_*pest_10*_, *t*_*pest_60*_, *t*_*pest_*900_, *t*_*pest_3600*_ or *t*_*pest_1h*_ were also not adjusted. Median values ($\overset{\sim }{i}$) are given with their interquartile range (IQR).

## Results

The control-test revealed neither a preference for any cardinal direction ($\overset{\sim }{i}$_*north*_ = 49.2%, IQR = 30.0–71.9%; Wilcoxon test vs. 50%: V = 72, p = 0.860; $\overset{\sim }{i}$_*west*_ = 51.2%, 29.9–61.9%; V = 74, p = 0.782; n = 16 in all tests) nor for the side orientated to the wall ($\overset{\sim }{i}$_*wall*_ = 39.4%, 26.6–69.6%; V = 63, p = 0.821) or to the other arena ($\overset{\sim }{i}$_*arena*_ = 41.9, 28.7–52.2%; V = 44, p = 0.231) over 24 hours.

**Fig 1:**
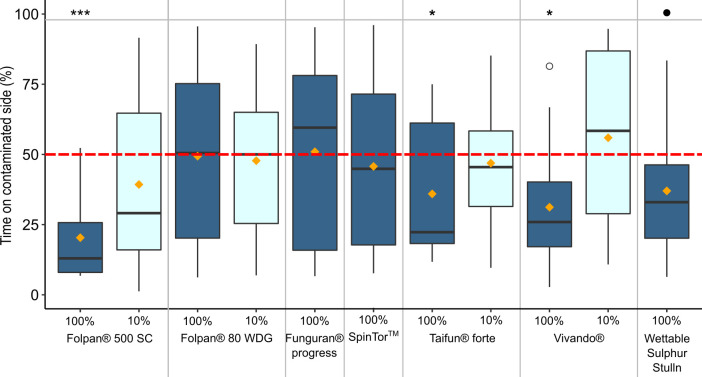
Boxplots showing the proportion of time a toad spent on the contaminated side of an arena over 24 hours for each tested formulation and concentration (*t*_*pest*_ in percentage; dark blue = 100% of the maximum recommended field rate (FR_max_), light blue = 10% of FR_max_). In each boxplot, the boundaries of the box are the 25^th^ and 75^th^ percentiles and the whiskers correspondent to the lowest and largest value no further than 1.5 times from the 25^th^ and 75^th^ percentiles away. Data points beyond the whiskers are shown as unfilled circles. Median values are presented as horizontal lines and orange diamonds show the mean values. Significant difference from a random choice (50%; red dotted line): ●: p adj. < 0.1; *: p adj. < 0.05; ***: p adj. < 0.001. P-values from tests with the same concentration were adjusted using the FDR. N = 16 per choice-test.

The animals spent on average less than 50% of their time on the contaminated side of the arena in all tested formulations at FR_max_ ($\overset{\sim }{i}$_*pest*_ < 50%; Fig 1 and Table 2), with the exception of Funguran® progress and Folpan® 80 WDG. Avoidance was significant for Folpan® 500 SC, Vivando® and Taifun® forte (Table 2). There was also a trend to avoid the contaminated side for Wettable Sulphur Stulln (p adj. = 0.068, but p = 0.039 without FDR; Table 2). No significant avoidance was observed when using a concentration of 10% of FR_max_ in any formulation (Table 2).

**Table 2:** Proportion of time a toad spent on the contaminated side of an arena (*t*_*pest*_) for each tested formulation and concentration (10% or 100% of the maximum recommended field rate; FR_max_) and results from two-sided one-sample Wilcoxon signed-rank tests that were used to compare *t*_*pest*_ to a theoretical value of 50% that can be expected from a random side choice.

Formulation	% of FR_max_	Time on contaminated side (%) *t*_*pest*_				Wilcoxon-Test—compared to 50%		
		Median	IQR	Range	Mean	V	p	p adj.
Folpan® 500 SC	100	13.0	8.0–25.7	6.8–52.3	20.3	2	**< 0.001**	**< 0.001**
Folpan® 500 SC	10	29.1	16.0–64.7	1.2–91.6	39.3	41	0.175	0.701
Folpan® 80 WDG	100	50.5	20.2–75.2	6.2–95.6	49.4	67	0.980	0.980
Folpan® 80 WDG	10	50.0	25.4–65.0	6.9–89.3	47.8	62	0.782	0.782
Funguran® progress	100	59.5	15.9–78.1	6.6–95.3	50.9	65	0.900	0.980
SpinTor^TM^	100	44.9	17.8–71.5	7.7–96.1	45.7	58	0.632	0.885
Taifun® forte	100	22.3	18.3–61.2	11.7–75.0	35.9	22	**0.016**	**0.036**
Taifun® forte	10	45.5	31.5–58.4	9.6–85.2	46.9	57	0.597	0.782
Vivando®	100	25.9	17.1–40.2	2.8–81.4	31.2	17	**0.006**	**0.022**
Vivando®	10	58.4	28.9–86.8	10.8–94.7	55.9	86	0.375	0.751
Wettable Sulphur Stulln	100	33.0	20.1–46.3	6.4–83.5	37.0	28	**0.039**	0.068

P-values from tests with the same concentration were adjusted using the FDR. Significant values are presented in bold. N = 16 per choice-test.

**Fig 2:**
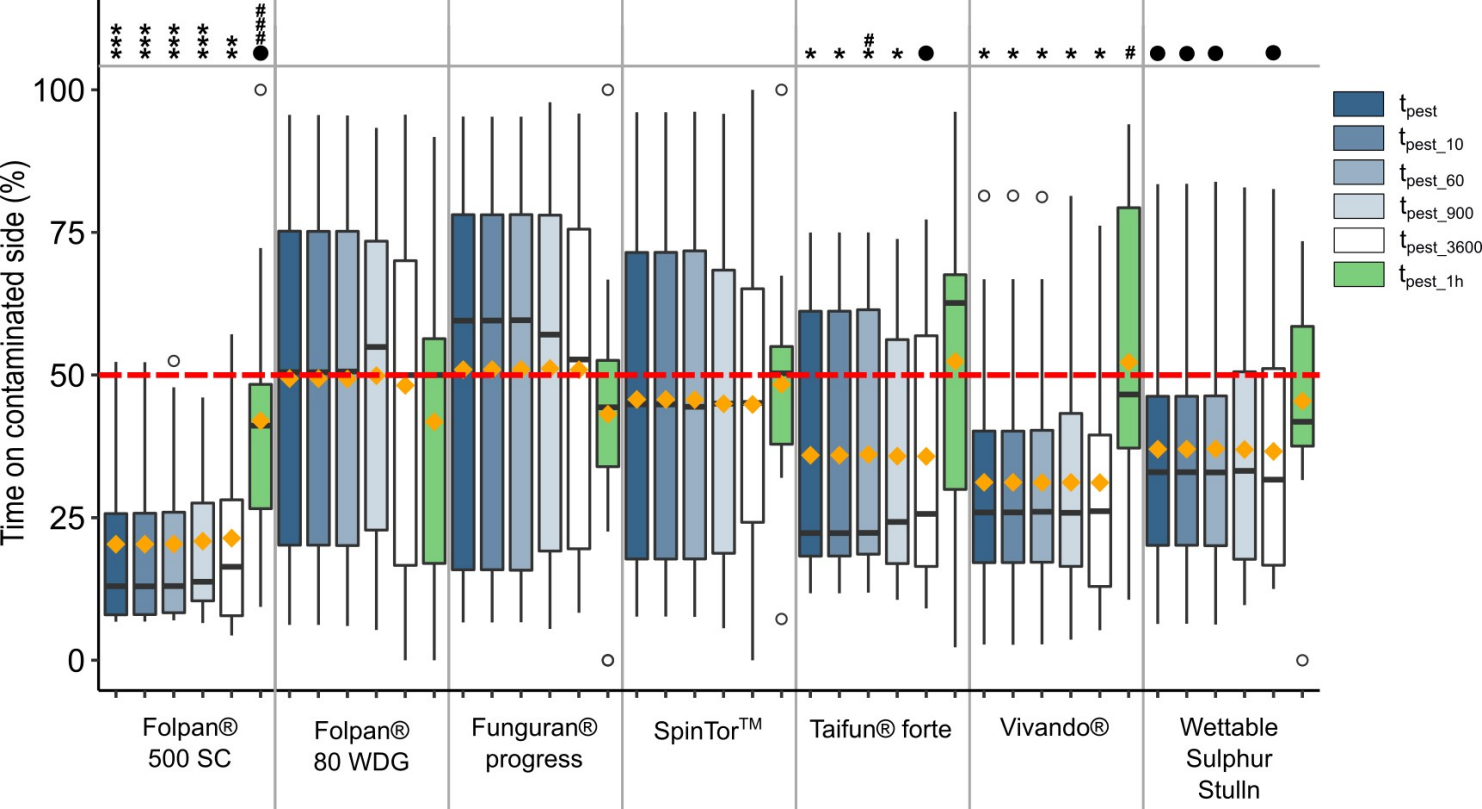
Boxplots showing the proportion of time a toad spent on the contaminated side of an arena for each tested formulation at the maximum recommended field rate (FR_max_) and for different sampling intervals. For the calculation of *t*_*pest*_ all data over 24 hours were used. For *t*_*pest_10*_, *t*_*pest_60*_, *t*_*pest_900*_ and *t*_*pest_3600*_ only one side choice every 10, 60, 900 and 3,600 seconds, respectively, were considered. *t*_*pest*_1h_ contains only data from the first hour of an experimental run. In each boxplot, the boundaries of the box are the 25^th^ and 75^th^ percentiles and the whiskers correspondent to the lowest and largest value no further than 1.5 times from the 25^th^ and 75^th^ percentiles away. Data points beyond the whiskers are shown as unfilled circles. Median values are presented as horizontal lines and orange diamonds show the mean values. Significant difference from a random choice (50%; red dotted line): ●: p adj. < 0.1; *: p adj. < 0.05; **: p adj. < 0.01; ***: p adj. < 0.001. P-values from tests with the same sampling interval were adjusted using the FDR. Significant differences compared to *t*_*pest*_: # = p < 0.05; ### = p < 0.001. N = 16 per choice-test.

The reduction of the sampling interval did not result in significant differences in the proportion of time spent on the contaminated side (all p > 0.144 when testing *t*_*pest*_ against *t*_*pest_10*_, *t*_*pest_60*_, *t*_*pest_*900_ or *t*_*pest_3600*_; Fig 2 and Table 3), with the exception of *t*_*pest_60*_ in Taifun® forte. Also the overall trend to prefer one side stayed the same when comparing *t*_*pest_10*_, *t*_*pest_60*_, *t*_*pest_*900_ or *t*_*pest_3600*_ against a random side choice (50%, Table 3). However, without adjusting the p-values with the FDR, significance was lost for Wettable Sulphur Stulln at a sample interval of one sample every 15 minutes (*t*_*pest_900*_), and for Taifun® forte at a sample interval of one sample every hour (*t*_*pest_3600*_) when using the FDR (Table 3). Restricting the study time to the first hour of the test (*t*_*pest_1h*_) resulted in significant differences to *t*_*pest*_ in Folpan® 500 SC and Vivando® (Fig 2 and Table 3). When testing *t*_*pest_1h*_ against a random side choice no significant avoidance of the contaminated side was found for any tested formulation.

**Table 3:** Proportion of time a toad spent on the contaminated side of an arena for each tested formulation at the maximum recommended field rate (FR_max_) and for different sampling intervals.

Formulation	Sampling interval	Time on contaminated side (%) *t*_*pest*_				Wilcoxon-Test—comp. to 50%			Wilcoxon-Test—comp. to *t*_*pest*_	
		Median	IQR	Range	Mean	V	p	p adj.	V	p
Folpan® 500 SC	*t*_*pest*_	13.0	8.0–25.7	6.8–52.3	20.3	2	**< 0.001**	**< 0.001**	not tested	
	*t*_*pest_10*_	13.0	8.0–25.8	6.8–52.2	20.3	2	**< 0.001**	**< 0.001**	65	0.900
	*t*_*pest_60*_	13.0	8.3–26.0	7.0–52.5	20.4	2	**< 0.001**	**< 0.001**	56	0.562
	*t*_*pest_900*_	13.8	10.4–27.6	6.5–46.1	20.9	0	**< 0.001**	**< 0.001**	52	0.433
	*t*_*pest_3600*_	16.4	7.8–28.1	4.3–57.1	21.4	3	**< 0.001**	**0.006**	54	0.495
	*t*_*pest_1h*_	41.1	26.6–48.4	9.4–100.0	42.0	34	0.083	0.583	7	**< 0.001**
Folpan® 80 WDG	*t*_*pest*_	50.5	20.2–75.2	6.2–95.6	49.4	67	0.980	0.980	not tested	
	*t*_*pest_10*_	50.5	20.2–75.2	6.2–95.6	49.4	67	0.989	0.980	94	0.193
	*t*_*pest_60*_	50.6	20.1–75.2	6.0–95.5	49.3	67	0.980	0.980	80	0.562
	*t*_*pest_900*_	54.9	22.8–73.5	5.3–93.3	49.9	69	0.980	1.000	54	0.495
	*t*_*pest_3600*_	50.0	16.6–70.0	0.0–95.7	48.2	47	0.754	0.879	84	0.433
	*t*_*pest_1h*_	50.0	17.0–56.4	0.0–91.7	41.8	46	0.454	0.835	78	0.330
Funguran® progress	*t*_*pest*_	59.5	15.9–78.1	6.6–95.3	50.9	65	0.900	0.980	not tested	
	*t*_*pest_10*_	59.6	15.9–78.1	6.6–95.3	51.0	65	0.900	0.980	42	0.193
	*t*_*pest_60*_	59.6	15.8–78.1	6.7–95.3	51.0	65	0.980	0.980	44	0.231
	*t*_*pest_900*_	57.1	19.2–78.0	5.5–97.8	51.1	68	1.000	1.000	63	0.821
	*t*_*pest_3600*_	52.7	19.5–75.6	8.3–95.8	50.9	69	0.980	0.979	73	0.821
	*t*_*pest_1h*_	44.4	33.9–52.6	0.0–100.0	43.1	41	0.170	0.596	99	0.117
SpinTor^TM^	*t*_*pest*_	44.9	17.8–71.5	7.7–96.1	45.7	58	0.632	0.885	not tested	
	*t*_*pest_10*_	44.9	17.8–71.5	7.7–96.1	45.7	58	0.632	0.885	72	0.860
	*t*_*pest_60*_	44.4	17.8–71.7	7.6–96.2	45.7	56	0.562	0.789	85	0.404
	*t*_*pest_900*_	45.0	18.8–68.4	5.6–95.8	45.0	56.5	0.570	0.797	90	0.274
	*t*_*pest_3600*_	45.1	24.2–65.1	0.0–100.0	44.8	53.5	0.469	0.657	82	0.495
	*t*_*pest_1h*_	50.3	37.9–55.0	7.2–100.0	48.4	57	0.597	0.835	61	0.744
Taifun® forte	*t*_*pest*_	22.3	18.3–61.2	11.7–75.0	35.9	22	**0.016**	**0.036**	not tested	
	*t*_*pest_10*_	22.3	18.3–61.2	11.7–75.0	36.0	22	**0.016**	**0.036**	39	0.144
	*t*_*pest_60*_	22.3	18.6–61.5	11.9–75.0	36.1	22	**0.016**	**0.036**	23	**0.018**
	*t*_*pest_900*_	24.3	17.0–56.2	10.6–73.9	35.8	24	**0.021**	**0.050**	68	1.000
	*t*_*pest_3600*_	25.7	16.5–56.9	9.1–77.3	35.7	25.5	**0.030**	0.067	71	0.900
	*t*_*pest_1h*_	62.6	30.0–67.6	2.3–96.1	52.4	71	0.900	0.900	35	0.093
Vivando®	*t*_*pest*_	25.9	17.1–40.2	2.8–81.4	31.2	17	**0.006**	**0.022**	not tested	
	*t*_*pest_10*_	25.9	17.1–40.2	2.7–81.4	31.2	17	**0.006**	**0.022**	87	0.348
	*t*_*pest_60*_	26.0	17.2–40.3	2.8–81.2	31.2	17	**0.006**	**0.022**	71	0.900
	*t*_*pest_900*_	25.9	16.5–43.3	3.6–81.4	31.2	19	**0.009**	**0.032**	68	1.000
	*t*_*pest_3600*_	26.1	12.9–39.5	5.3–76.2	31.1	17	**0.006**	**0.022**	53	0.464
	*t*_*pest_1h*_	46.6	37.2–79.3	10.6–94.0	52.2	49	0.839	0.900	15	**0.033**
Wettable Sulphur Stulln	*t*_*pest*_	33.0	20.1–46.3	6.4–83.5	37.0	28	**0.039**	0.068	not tested	
	*t*_*pest_10*_	33.0	20.2–46.3	6.4–83.5	37.0	28	**0.039**	0.068	53	0.464
	*t*_*pest_60*_	32.9	20.1–46.3	6.3–83.9	37.1	29	**0.044**	0.078	53	0.464
	*t*_*pest_900*_	32.5	17.7–50.6	9.7–82.9	36.9	27	0.065	0.114	78	0.632
	*t*_*pest_3600*_	31.7	16.7–51.5	12.5–82.6	36.6	23	**0.038**	0.067	77	0.669
	*t*_*pest_1h*_	41.8	37.5–58.5	0.0–73.5	45.4	48	0.524	0.835	38	0.229

For each formulation and sampling interval results from statistical tests that were used to compare *t*_*pest*_, *t*_*pest_10*_, *t*_*pest_60*_, *t*_*pest_*900_, *t*_*pest_3600*_ or *t*_*pest_1h*_ against a random side choice (50%) and to compare *t*_*pest*_ against *t*_*pest_10*_, *t*_*pest_60*_, *t*_*pest_*900_, *t*_*pest_3600*_ or *t*_*pest_1h*_ are given. When testing *t* against 50%, p-values from the same sampling interval were also adjusted using the FDR (p adj.). Significant values are presented in bold. N = 16 per choice-test.

**Fig 3:**
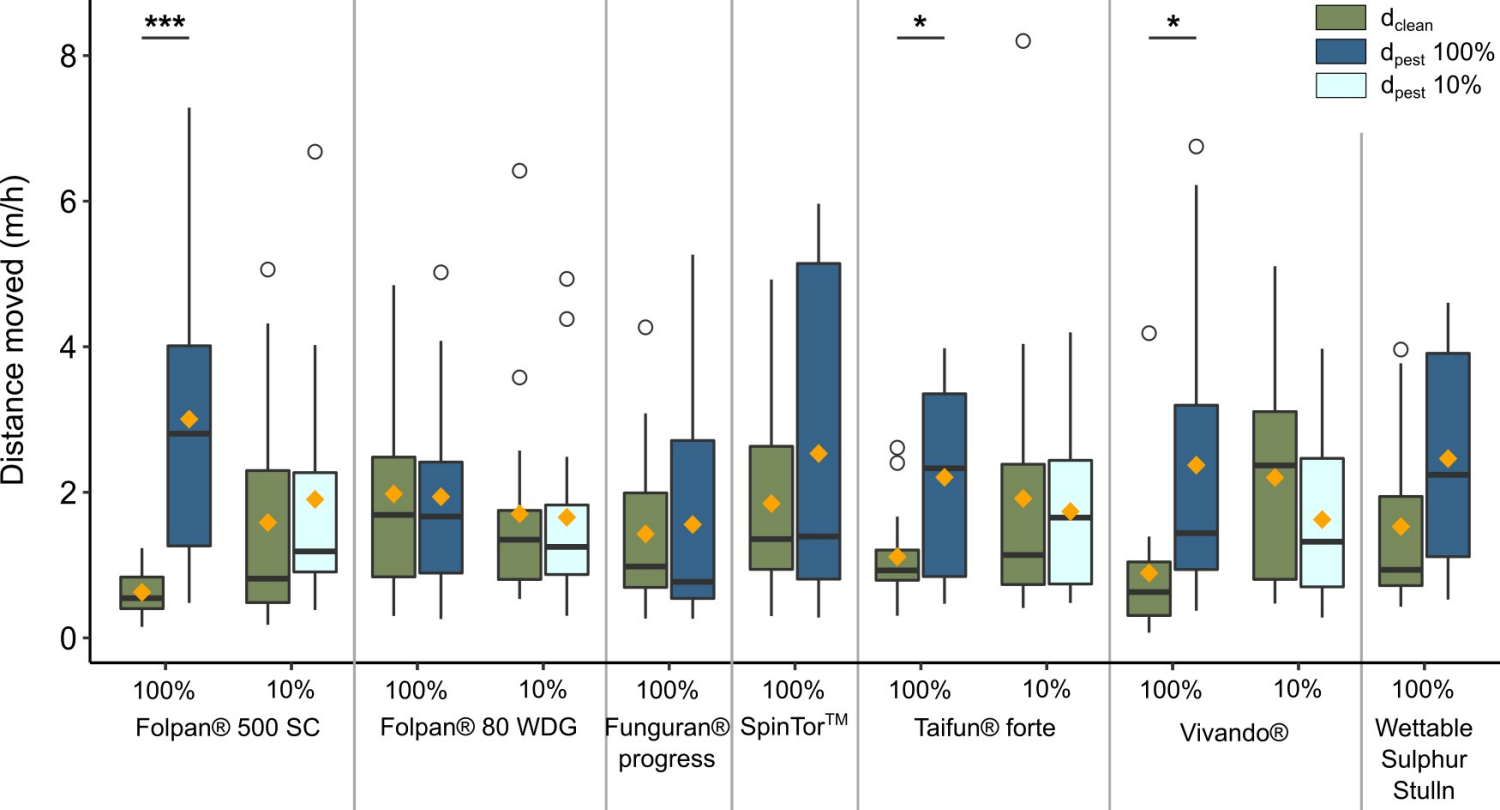
Boxplots showing the distance moved in meter per hour on the contaminated (*d*_*pest*_; dark blue = 100% of the maximum recommended field rate (FR_max_), light blue = 10% of FR_max_) and uncontaminated side (*d*_*clean*_; green) of an arena over 24 hours. In each boxplot, the boundaries of the box are the 25^th^ and 75^th^ percentiles and the whiskers correspondent to the lowest and largest value no further than 1.5 times from the 25^th^ and 75^th^ percentiles away. Data points beyond the whiskers are shown as unfilled circles. Median values are presented as horizontal lines and orange diamonds show the mean values. Significant difference between *d*_*pest*_ and *d*_*clean*_: *: p adj. < 0.05; ***: p adj. < 0.001. P-values from tests with the same concentration were adjusted using the FDR. N = 16 per choice-test.

In the three choice-tests in which we observed a significant difference between *t*_*pest*_ and a random side choice, also significant differences in the activity of the toads were found (Table 4). The median distance a toad moved on the contaminated side per hour was on average 5.1 times longer for Folpan® 500 SC, 2.3 times longer for Vivando®, and 2.5 times longer for Taifun® forte than the distance moved on the uncontaminated side. In all other choice-tests no activity differences were observed (Fig 3 and Table 4).

**Table 4:** Distances moved in meter per hour on the contaminated (pest; 10% or 100% of the of the maximum recommended field rate (FR_max_); *d*_*pest*_) and uncontaminated (clean; *d*_*clean*_) side of an arena and results from two-sided Wilcoxon signed-rank tests that were used to compare *d*_*pest*_ and *d*_*clean*_.

Formulation	% of FR_max_	Side	Distance moved (m/h)				Wilcoxon-Test - clean vs. pest		
			Median	IQR	Range	Mean	V	p	p adj.
Folpan® 500 SC	100	Clean	0.55	0.40–0.84	0.15–1.23	0.63	0	**< 0.001**	**< 0.001**
		Pest	2.81	1.26–4.01	0.48–7.28	3.01			
Folpan® 500 SC	10	Clean	0.81	0.49–2.30	0.18–5.06	1.58	58	0.632	0.701
		Pest	1.19	0.91–2.27	0.38–6.68	1.90			
Folpan® 80 WDG	100	Clean	1.69	0.84–2.48	0.30–4.85	198	72	0.860	0.860
		Pest	1.67	0.89–2.42	0.26–5.02	1.94			
Folpan® 80 WDG	10	Clean	1.35	0.80–1.75	0.53–6.42	1.70	70	0.934	0.782
		Pest	1.25	0.87–1.83	0.30–4.93	1.66			
Funguran® progress	100	Clean	0.98	0.69–1.99	0.27–4.27	1.43	64	0.860	0.860
		Pest	0.77	0.54–2.71	0.26–5.26	1.56			
SpinTor^TM^	100	Clean	1.36	0.94–2.63	0.30–4.92	1.85	52	0.433	0.606
		Pest	1.39	0.81–5.14	0.28–5.97	2.53			
Taifun® forte	100	Clean	0.93	0.79–1.21	0.30–2.61	1.11	24	**0.021**	**0.050**
		Pest	2.33	0.84–3.35	0.47–3.98	2.21			
Taifun® forte	10	Clean	1.14	0.73–2.39	0.41–8.20	1.91	66	0.934	0.782
		Pest	1.65	0.74–2.44	0.48–4.20	1.74			
Vivando®	100	Clean	0.63	0.31–1.04	0.07–4.19	0.89	17	**0.006**	**0.022**
		Pest	1.44	0.94–3.20	0.37–6.75	2.37			
Vivando®	10	Clean	2.37	0.80–3.11	0.47–5.11	2.20	86	0.376	0.751
		Pest	1.32	0.70–2.47	0.28–3.97	1.63			
Wettable Sulphur Stulln	100	Clean	0.94	0.72–1.94	0.43–3.96	1.53	31	0.058	0.101
		Pest	2.24	1.12–3.91	0.53–4.61	2.46			

P-values from tests with the same concentration were adjusted using the false discovery rate. Significant values are presented in bold. N = 16 per choice-test.

## Discussion

Based on over 2,300 hours of video recordings, we found evidence of an avoidance behavior of common toad juveniles for three out of seven tested pesticide formulations at maximum recommended field rates. For one other formulation a trend for avoidance could be observed. As we could exclude the presence of external cues or a cardinal direction with the control-test, the observed side choice can be traced back to the pesticide. Overspraying the surface with the maximum recommended field rate represents a worst-case scenario. Fungicides and insecticides are usually applied directly on the plant, resulting in an interception by the crop and therefore a reduced concentration on the ground [50]. However, especially fungicides are applied several times per year with short time periods between applications and often as mixtures of several formulations [8, 46, 51], increasing the overall soil pesticide load. Further, herbicides like the tested glyphosate formulation Taifun® forte are usually directly applied on the ground. Therefore, contamination of the soil with the field rate is a worst-case, but still realistic scenario.

To avoid a contaminated surface, toads have to be able to detect the contamination. As the used formulations did not dye the silica sand, visual detection is unlikely. Therefore, the detection is likely to be related to olfactory or somatosensory perception, or internal mechanisms like a metabolic response that triggers a purpose-orientated behavior and presupposes the uptake of the substance. As amphibians have a highly permeable skin [23], an uptake is possible when they come in contact with contaminated soil [11, 12]. However, as shown for the common wall lizard (*Podarcis muralis*) [52], the metabolic response might be time-delayed, making it unlikely for the toad to link the metabolic response to the pesticide exposure and to subsequently react with an avoidance of a contaminated surface. In Storrs Méndez et al. [11] an uptake of atrazine was demonstrated for the American toad (*B*. *americanus*), but even after 60 hours, no avoidance behavior was observed. Amphibians have a good olfactory perception and use chemical cues for example during courtship [21] or for orientation [22]. Juvenile *B*. *bufo* are able to perceive and recognize olfactory cues from different sources, e.g. lake water [53]. Farabaugh and Nowakowski [54] demonstrated that the strawberry poison frog (*Oophaga pumilio*) can use olfactory cues to detect the glyphosate herbicide Roundup^TM^. Therefore, the detection of olfactory cues from contaminated surfaces might be possible. However, it remains unknown if the differentiation of contaminated and uncontaminated areas based on olfactory cues is possible in an arena with a diameter of only 20 cm like in our setup. Compared to the olfactory perception, the somatosensory perception might be more independent from the dimensions of the arena and the contaminated and uncontaminated areas. The active ingredient or at least one of the additives of all three avoided pesticide formulations, as well as of Wettable Sulphur Stulln, where a trend to avoidance could be found, are classified as "Causes severe skin burns and eye damage" or "Causes skin irritation". This is not the case for the other tested formulations, even though Folpan® 80 WDG is classified as "May cause an allergic skin reaction" (Tab. 1). Therefore, these classifications could be an indicator for an avoidance behavior. However, some classified additives can only be found in small amounts in the formulation (e.g. < 0.1% 3-Benzisothiazolinon in Folpan® 500 SC) and also the number of tested formulations is too low to draw any general conclusion. Therefore, the physiological mechanisms of the avoidance remain unknown, and could also be different between formulations.

Interestingly, we found a significant avoidance of Folpan® 500 SC, but not of Folpan® 80 WDG. Both formulations have the same active ingredient folpet and were tested in their maximum recommended field rate, which results in a comparable amount of the active ingredient (1.20 and 1.28 kg a.i/ha). Therefore, toads might not be able to detect folpet. Observed differences in the avoidance cannot be explained by the active ingredient, but might be the result of additives in the formulation. Additives change the characteristics of the formulation and several studies showed that they can enhance or decrease toxic effects [17, 55, 56]. Folpet is classified as "May cause an allergic skin reaction", but 3-benzisothiazolinone, an additive only in Folpan® 500 SC, is also classified as "Causes skin irritation", which might affect the avoidance behavior. Individuals tested on Folpan® 500 SC were captured in the beginning of August, while individuals used for Folpan ® 80 WDG were captured in the beginning of September, so were about one month older and also differed in their body weight (S1 Table). It cannot be ruled out that these differences influenced the behavior during the tests and therefore caused the contrasting results among the two folpet formulations. Due to the variability between experimental runs in weight/size and age of the individuals, but also in the time the toads were kept in the cages before the experiment or the exact starting time of the experiment (S1 Table), comparisons among experimental runs can only be made with caution. Differences in the age, but also differences in the habitat use (i.e. the time spent in vineyards) might also come with differences in the exposure to pesticides before the experimental run. As each pesticide was tested only once at 10 or 100% of FR_max_, general conclusion if and how all these factors affect the avoidance behavior cannot be stated. Thus, their combined effects should be examined in future studies in detail.

In previous studies, amphibians were able to detect and therefore avoid pesticides in the laboratory on artificial surfaces like filter paper, but usually not on more natural soils. Hatch et al. [29] conducted choice tests with urea, which is used as fertilizer in agriculture and forestry. Juvenile western toads (*Bufo boreas*) and cascades frogs (*Rana cascadae*) avoided urea-dosed paper towels in an arena experiment, but showed no preference when a natural substrate was used. In contrast, Gaglione et al. [30] found avoidance of urea both on contaminated filter paper as well as commercial top soil for the red-backed salamander (*Plethodon cinereus*). Gertzog et al. [31] showed that *P*. *cinereus* also avoids filter paper contaminated with three different herbicide formulations. Also Iberian newts (*Lissotriton boscai*, formerly *Triturus boscai*) avoid filter paper dosed with the fertilizer ammonium nitrate [32]. Storrs Méndez et al. [11] conducted choice tests with the herbicide atrazine on soil. Although atrazine was absorbed by juvenile American toads (*Bufo americanus*), no avoidance could be detected. In terms of environmental realism, we rank the silica sand used in our study system as intermediate between studies with contaminated filter paper and natural soil. Although loamy to sandy soils can be found in vineyards, organic components are completely lacking in the sand we used, which is unrealistic for natural soils. The organic matter content of soils affects the bioavailability, uptake and thus bioaccumulation of pesticides by amphibians [57], and could therefore also play a role in the avoidance behavior. We chose the silica sand mainly because of its coloration, as its brightness increased the contrast to the dark toads. Most natural soils would have been darker, thus decreasing the contrast to the experimental animal and increasing the probability of errors during the automatic detection of the toads in the arenas by EthoVision^®^ XT. Natural soils could be tested when side choice is determined manually without a tracking software. However, this would require the reduction of the sampling interval. A reduction to every 3,600 seconds (= 1 hour; resulting in 24 frames when filming for 24 hours) or 900 seconds (= 15 minutes; 360 frames over 24 hours) would allow determining the side choice manually without a tracking software. The reduction to one data point every 10 or 60 seconds would only allow to speed up the, in some cases long-lasting, analysis with the tracking software. In general, the reduction can be expected to have only little effect on the proportion of time spent on a side, as differences presuppose that toads are very active and are changing the side frequently. However, in cases where the avoidance behavior is only weak, also small differences might result in an increased probability of false-positive or false-negative results. In our study, a weak avoidance behavior was observed for Taifun® forte at a sample interval of one sample per hour (*t*_*pest_3600*_; p = 0.030). Nevertheless, in a screening of several pesticide formulations, one has to consider the probability of a type I error, and thus adjust the p-values of statistical tests, which resulted in the loss of significance in Taifun® forte (*t*_*pest_3600*_). P-value adjustment also resulted in p-values above the criterion of significance (0.05) for *t*_*pest*_ and all subsamplings of *t*_*pest*_ when testing Wettable Sulphur Stulln. Thus, the same avoidance response of the toads to the pesticide was found for all sample intervals. However, when solely regarding Wettable Sulphur Stulln without using the FDR, a significant avoidance behavior was found for *t*_*pest*_, *t*_*pest_10*_, *t*_*pest_60*_, and *t*_*pest_3600*_, but not for *t*_*pest_900*_. Thus, both a sampling interval of one sample per 15 min and one sample per hour could have led to an overlooked avoidance behavior in one pesticide formulation. When the data was limited to the first hour of an experiment, no avoidance behavior could be detected for any tested pesticide. Some toads did not move at all during the first hour, underlining the importance of a prolonged acclimatization period. Future studies on amphibian avoidance behavior should be aware of these problems and should not neglect cases where no significance, but a trend is found, e.g. when it comes to choosing formulations for a higher-tier-assessment. As we found high variability in the behavior of tested toads, we would further recommend to increase the number of replicates, if possible.

Alterations of the movement behavior after pesticide exposure are well known for amphibians. An abnormal swimming behavior and a decreased activity of larvae can often be observed [37, 58], whereby such alterations are usually induced by the neurotoxicity of the pesticide [59]. In our study, differences in the distance moved per hour on the contaminated *versus* the uncontaminated side might be rather linked to the avoidance behavior, in the sense that toads might have avoided resting on the contaminated side for longer periods. Consequently, we found increases in the moved distance on the contaminated side in the choice-tests with Folpan® 500 SC, Vivando® and Taifun® forte. In general, most studies on amphibian behavioral response to pesticides are focusing on the larval stages in an aquatic environment [37], which corresponds to the underrepresentation of terrestrial life stages in ecotoxicological studies [60]. Considering the high toxicity of some pesticides for terrestrial amphibians [16–18], the numerous studies on effects in the aquatic habitat [37] and the effects of pesticides on the behavior of other ectothermic groups like lizards [61], it is likely that pesticides can also alter the behavior of terrestrial amphibians. However, most studies on the effects of pesticides on terrestrial amphibians did not find evidence for behavior alterations (see review in [60]). One explanation might be a lack of standardized methods and adequate endpoints to study these alterations. To our surprise, we found no ecotoxicological study in which automatic video tracking of exposed individuals was used in terrestrial amphibians, although this method is often used in a variety of taxa like bees [62], green lacewings [63] or mice [64] and also for aquatic amphibian larvae [65, 66]. This method might provide informative endpoints in future terrestrial amphibian studies in an upcoming pesticide risk assessment for amphibians. The setup we used, which is based on a Raspberry Pi, might help researchers to study these aspects, as it allows the filming of multiple individuals in parallel and it is a simple, freely configurable and affordable alternative to specialized video equipment. Besides highly professional tracking software like EthoVision there is also a rising number of open-source, freely available alternatives [67].

We detected avoidance of three out of seven tested pesticide formulations at 100% of FR_max,_ and no avoidance when using a concentration of 10% of FR_max_ in any formulation. As agriculture with frequent pesticide applications is the dominant type of land use in many regions, an avoidance might contribute to a chemical landscape fragmentation. Landscape fragmentation can lead to reduced gene flow between, and as a result, reduced fitness of amphibian populations [68]. On the other hand, the lack of avoidance behavior in the other tested formulations might increase the pesticide exposure risk of amphibians in agricultural landscapes, which could lead to sublethal [13–15] and lethal effects, even at field rates of 10% [17]. Therefore, we conclude that a heterogeneous landscape with green corridors between populations and different habitat types is needed so that contaminated areas can be avoided without leading to a fragmentation of the landscape. Future studies on behavior choice tests should consider adult individuals, natural soils with different contents of organic matter as well as soils that have been oversprayed not directly before the test allowing adsorption to soil to represent other potential scenarios. Testing individuals from uncontaminated populations would help to understand whether the avoidance is an evolved adaption. Future tests should also reflect realistic application sequences with mixtures of multiple pesticides [69]. Last but not least, field studies are needed to verify results from laboratory studies under realistic conditions.

## Supporting information

S1 Table Detailed information about each choice test. The table includes the date when the toads were captured, the date of the experimental run, the times when the first and the last toad were placed in the test arenas, the mean weight with its standard deviation (SD) of the toads used in each test as well the proportion of the total time a toad spent in a 2.5 cm wide area at the border between the contaminated and uncontaminated side of an area (buffer zone). Positions of toads in the buffer zone were excluded when analyzing the avoidance behavior. FR_max_ is the maximum recommended field rate of a formulation.
(DOCX)

S1 Fig Map of the study area. The points show the location of the pond where the individuals for the experimental runs were captured (blue; "Pond"), the location where insects for feeding of the toads were captured (yellow; "Meadow") and the location where soil, moss and leaves were collected to equip the outdoor net cages (red; "Palatinate Forest"). Reprinted from www.lvermgeo.rlp.de under a CC BY license, with permission from GeoBasis-DE / LVermGeoRP2020, original copyright 2020.
(JPG)

S2 Fig Glass petri dish filled with silica sand before the pesticide application. One side is covered with laminated paper semicircles to prevent contamination of the clean side during the application process.
(JPG)

S3 Fig Two test arenas with experimental animals in a test chamber right before test start. Arenas are covered with a polyamide fabric.
(JPG)

S4 Fig Photo of the camera module (SC15; Kuman Ltd., Shenzhen, China) that was used to record the toads and used LED lights. The camera was attached to a Raspberry Pi (Raspberry Pi 3 Model B; Raspberry Pi Foundation, Cambridge, UK).
(JPG)

S5 Fig Screenshot of a video recorded during one of the choice tests showing experimental animals in their arena with the visualization of the track of the animals from EthoVision® XT. (PNG)

## References

[1] Eurostat. Land use overview by NUTS 2 regions—Agriculture [Internet]. 2020 [cited 18 Jan 2020]. Available: http://appsso.eurostat.ec.europa.eu/nui/show.do?query=BOOKMARK_DS-669621_QID_59255FAF_UID_-3F171EB0&layout=TIME,C,X,0;GEO,L,Y,0;UNIT,L,Z,0;LANDUSE,L,Z,1;INDICATORS,C,Z,2;&zSelection=DS-669621INDICATORS,OBS_FLAG;DS-669621LANDUSE,LUA;DS-669621UNIT,PC;&rankN

[2] Eurostat. Pesticide sales [Internet]. 2020 [cited 18 Jan 2020]. Available: https://appsso.eurostat.ec.europa.eu/nui/show.do?query=BOOKMARK_DS-382683_QID_7670BBE_UID_-3F171EB0&layout=PESTICID,L,X,0;TIME,C,X,1;GEO,L,Y,0;UNIT,L,Z,0;INDICATORS,C,Z,1;&zSelection=DS-382683UNIT,KG;DS-382683INDICATORS,OBS_FLAG;&rankName1=UNIT_1_2_-1_2&r

[3] Hvězdová M, Kosubová P, Košíková M, Scherr KE, Šimek Z, Brodský L, et al Currently and recently used pesticides in Central European arable soils. Sci Total Environ. 2018;613–614: 361–370. 10.1016/j.scitotenv.2017.09.049 28917175

[4] Silva V, Mol HGJ, Zomer P, Tienstra M, Ritsema CJ, Geissen V. Pesticide residues in European agricultural soils–A hidden reality unfolded. Sci Total Environ. 2019;653: 1532–1545. 10.1016/j.scitotenv.2018.10.441 30759587

[5] Beja P, Alcazar R. Conservation of Mediterranean temporary ponds under agricultural intensification: An evaluation using amphibians. Biol Conserv. 2003;114: 317–326. 10.1016/S0006-3207(03)00051-X

[6] Lenhardt PP, Schäfer RB, Theissinger K, Brühl CA. An expert-based landscape permeability model for assessing the impact of agricultural management on amphibian migration. Basic Appl Ecol. 2013;14: 442–451. 10.1016/j.baae.2013.05.004

[7] Knutson MG, Richardson WB, Reineke DM, Gray BR, Parmelee JR, Weick SE. Agricultural ponds support amphibian populations. Ecol Appl. 2004;14: 669–684. 10.1890/02-5305

[8] Leeb C, Brühl C, Theissinger K. Potential pesticide exposure during the post-breeding migration of the common toad (Bufo bufo) in a vineyard dominated landscape. Sci Total Environ. 2020;706: 134430 10.1016/j.scitotenv.2019.134430 31855631

[9] Lenhardt PP, Brühl CA, Berger G. Temporal coincidence of amphibian migration and pesticide applications on arable fields in spring. Basic Appl Ecol. 2014;16: 54–63. 10.1016/j.baae.2014.10.005

[10] Berger G, Graef F, Pfeffer H. Glyphosate applications on arable fields considerably coincide with migrating amphibians. Sci Rep. 2013;3: 2622 10.1038/srep02622 24018602 PMC6505567

[11] Storrs Méndez SI, Tillitt DE, Rittenhouse TAG, Semlitsch RD. Behavioral response and kinetics of terrestrial atrazine exposure in American toads (Bufo americanus). Arch Environ Contam Toxicol. 2009;57: 590–597. 10.1007/s00244-009-9292-0 19198750

[12] Van Meter RJ, Glinski DA, Henderson WM, Garrison AW, Cyterski M, Purucker ST. Pesticide uptake across the amphibian dermis through soil and overspray exposures. Arch Environ Contam Toxicol. 2015;69: 545–556. 10.1007/s00244-015-0183-2 26135301

[13] Van Meter RJ, Glinski DA, Purucker ST, Henderson WM. Influence of exposure to pesticide mixtures on the metabolomic profile in post-metamorphic green frogs (Lithobates clamitans). Sci Total Environ. 2018;624: 1348–1359. 10.1016/j.scitotenv.2017.12.175 29929247 PMC6020053

[14] Franco-Belussi L, Moreno L, Tripole S, Natale GS, Pérez-Iglesias JM, de Oliveira C. Effects of glyphosate on hepatic tissue evaluating melanomacrophages and erythrocytes responses in neotropical anuran Leptodactylus latinasus. Environ Sci Pollut Res. 2016;23: 9852–9861. 10.1007/s11356-016-6153-z 26856864

[15] Ezemonye L, Tongo I. Sublethal effects of endosulfan and diazinon pesticides on glutathione-S-transferase (GST) in various tissues of adult amphibians (Bufo regularis). Chemosphere. 2010;81: 214–217. 10.1016/j.chemosphere.2010.06.039 20609459

[16] Belden J, McMurry S, Smith L, Reilley P. Acute toxicity of fungicide formulations to amphibians at environmentally relevant concentrations. Environ Toxicol Chem. 2010;29: 2477–2480. 10.1002/etc.297 20836054

[17] Brühl CA, Schmidt T, Pieper S, Alscher A. Terrestrial pesticide exposure of amphibians: An underestimated cause of global decline? Sci Rep. 2013;3: 1135 10.1038/srep01135 23350038 PMC3553602

[18] Relyea RA. The lethal impact of Roundup on aquatic and terrestrial amphibians. Ecol Appl. 2005;15: 1118–1124. 10.1890/04-1291

[19] Hua J, Morehouse NI, Relyea R. Pesticide tolerance in amphibians: Induced tolerance in susceptible populations, constitutive tolerance in tolerant populations. Evol Appl. 2013;6: 1028–1040. 10.1111/eva.12083 24187585 PMC3804236

[20] Wagner N, Veith M, Lötters S, Viertel B. Population and life-stage-specific effects of two herbicide formulations on the aquatic development of European common frogs (Rana temporaria). Environ Toxicol Chem. 2017;36: 190–200. 10.1002/etc.3525 27291460

[21] Treer D, Van Bocxlaer I, Matthijs S, Du Four D, Janssenswillen S, Willaert B, et al Love Is Blind: Indiscriminate Female Mating Responses to Male Courtship Pheromones in Newts (Salamandridae). PLoS One. 2013;8: 1–7. 10.1371/journal.pone.0056538 23457580 PMC3574087

[22] Sinsch U. Migration and orientation in anuran amphibians. Ethol Ecol Evol. 1990;2: 65–79. 10.1080/08927014.1990.9525494

[23] Kaufmann K, Dohmen P. Adaption of a dermal in vitro method to investigate the uptake of chemicals across amphibian skin. Environ Sci Eur. Springer Berlin Heidelberg; 2016;28: 1–13. 10.1186/s12302-016-0080-y 27752445 PMC5044961

[24] Llewelyn VK, Berger L, Glass BD. Percutaneous absorption of chemicals: Developing an understanding for the treatment of disease in frogs. J Vet Pharmacol Ther. 2016;39: 109–121. 10.1111/jvp.12264 26456710

[25] Smith PN, Cobb GP, Godard-Codding C, Hoff D, McMurry ST, Rainwater TR, et al Contaminant exposure in terrestrial vertebrates. Environ Pollut. 2007;150: 41–64. 10.1016/j.envpol.2007.06.009 17706848

[26] Vonesh JR, Buck JC. Pesticide alters oviposition site selection in gray treefrogs. Oecologia. 2007;154: 219–226. 10.1007/s00442-007-0811-2 17665220

[27] Takahashi M. Oviposition site selection: Pesticide avoidance by gray treefrogs. Environ Toxicol Chem. 2007;26: 1476–1480. 10.1897/06-511r.1 17665689

[28] Wagner N, Lötters S. Effects of water contamination on site selection by amphibians: experiences from an arena approach with European frogs and newts. Arch Environ Contam Toxicol. 2013;65: 98–104. 10.1007/s00244-013-9873-9 23377318

[29] Hatch AC, Belden LK, Scheessele E, Blaustein AR. Juvenile amphibians do not avoid potentially lethal levels of urea on soil substrate. Environ Toxicol Chem. 2001;20: 2328–2335. 10.1897/1551-5028(2001)020<2328:jadnap>2.0.co;2 11596767

[30] Gaglione CM, Meara EMO, Pence KL, Petterson AC, Smith GR, Rettig JE. Red-backed salamander, Plethodon cinereus (Green, 1818): avoidance of urea. Herpetol Notes. 2011;4: 275–277.

[31] Gertzog BJ, Kaplan LJ, Nichols D, Smith GR, Rettig JE. Avoidance of three herbicide formulations by Eastern Red-Backed Salamanders (Plethodon cinereus). Herpetol Conserv Biol. 2011;6: 237–241.

[32] Ortiz-Santaliestra ME, Marco A, Lizana M. Sensitivity and Behavior of the Iberian Newt, *Triturus boscai*, Under Terrestrial Exposure to Ammonium Nitrate. Bull Environ Contam Toxicol. 2005;75: 662–669. 10.1007/s00128-005-0803-z 16400545

[33] Salazar RD, Montgomery RA, Thresher SE, Macdonald DW. Mapping the relative probability of common toad occurrence in terrestrial lowland farm habitat in the United Kingdom. PLoS One. 2016;11: 1–14. 10.1371/journal.pone.0148269 26841108 PMC4739741

[34] Vos CC, Goedhart PW, Lammertsma DR, Spitzen-van der Sluijs AM. Matrix permeability of agricultural landscapes: an analysis of the common frog (Rana temporaria). Herpetol J. 2007;17: 174–182.

[35] Lenhardt PP, Brühl CA, Leeb C, Theissinger K. Amphibian population genetics in agricultural landscapes: does viniculture drive the population structuring of the European common frog (Rana temporaria)? PeerJ. 2017;5: e3520 10.7717/peerj.3520 28713651 PMC5508807

[36] Jean-Marc C, Pascal M, Alexandre B, Francis IN, Sandra G, Olivier L, et al Agricultural landscapes and the Loire River influence the genetic structure of the marbled newt in Western France. Sci Rep. 2018;8: 1–12. 10.1038/s41598-017-17765-5 30242196 PMC6155057

[37] Sievers M, Hale R, Parris KM, Melvin SD, Lanctôt CM, Swearer SE. Contaminant-induced behavioural changes in amphibians: A meta-analysis. Sci Total Environ. 2019;693: 133570 10.1016/j.scitotenv.2019.07.376 31369889

[38] Sillero N, Campos J, Bonardi A, Corti C, Creemers R, Crochet P-A, et al Updated distribution and biogeography of amphibians and reptiles of Europe. Amphibia-Reptilia. 2014;35: 1–31. 10.1163/15685381-00002935

[39] Agasyan A, Avisi A, Tuniyev B, Isailovic JC, Lymberakis P, Andrén C, et al Bufo bufo. IUCN Red List Threat Species 2009 2009;e.T54596A1. 10.2305/IUCN.UK.2009.RLTS.T54596A11159939.en

[40] Carrier J-A, Beebee TJ. Recent, substantial, and unexplained declines of the common toad *Bufo bufo* in lowland England. Biol Conserv. 2003;111: 395–399. 10.1016/S0006-3207(02)00308-7

[41] Petrovan SO, Schmidt BR. Volunteer conservation action data reveals large-scale and long-term negative population trends of a widespread amphibian, the common toad (Bufo bufo). PLoS One. 2016;11: e0161943 10.1371/journal.pone.0161943 27706154 PMC5051710

[42] Kyek M, Kaufmann PH, Lindner R. Differing long term trends for two common amphibian species (Bufo bufo and Rana temporaria) in alpine landscapes of Salzburg, Austria. PLoS One. 2017;12: e0187148 10.1371/journal.pone.0187148 29121054 PMC5679550

[43] Bonardi A, Manenti R, Corbetta A, Ferri V, Fiacchini D, Giovine G, et al Usefulness of volunteer data to measure the large scale decline of “common” toad populations. Biol Conserv. 2011;144: 2328–2334. 10.1016/j.biocon.2011.06.011

[44] Günther R, Geiger A. Erdkröte—Bufo bufo (Linnaeus, 1758) In: Günther R, editor. Die Amphibien und Reptilien Deutschlands. Heidelberg: Spektrum Verlag; 2009 pp. 274–302.

[45] Cushman SA. Effects of habitat loss and fragmentation on amphibians: A review and prospectus. Biol Conserv. 2006;128: 231–240. 10.1016/j.biocon.2005.09.031

[46] Roßberg D, Ipach R. Erhebungen zur Anwendung von Pflanzenschutzmitteln im Weinbau. J für Kult. 2015;67: 410–416. 10.5073/JFK.2015.12.03

[47] Council E. Regulation (EC) No 1272/2008 of the European Parliament and of the Council of 16 December 2008 on classification, labelling and packaging of substances and mixtures, amending and repealing Directives 67/548/EEC and 1999/45/EC, and amending Regulation (EC). Off J Eur Union. 2008;51: 1–1355.

[48] R Development Core Team. R: A language and environment for statistical computing. R Foundation for Statistical Computing. Vienna, Austria; 2020. 10.1111/j.1365-2621.1979.tb03829.x

[49] Benjamini Y, Hochberg Y. Controlling the false discovery rate: a practical and powerful approach to multiple testing. J R Stat Soc Ser B. 1995;57: 289–300. 10.2307/2346101

[50] Linders J, Mensink H, Stephenson G, Wauchope D, Racke K. Foliar interception and retention values after pesticide application. A proposal for standardized values for environmental risk assessment (Technical report). Pure Appl Chem. 2000;72: 2199–2218. 10.1351/pac200072112199

[51] Roßberg D. Erhebungen zur Anwendung von Pflanzenschutzmitteln in der Praxis im Jahr 2011. J für Kult. 2013;65: 141–151. 10.5073/JFK.2013.04.02

[52] Mingo V, Leeb C, Fahl A-K, Lötters S, Brühl C, Wagner N. Validating buccal swabbing as a minimal-invasive method to detect pesticide exposure in squamate reptiles. Chemosphere. 2019; 10.1016/j.chemosphere.2019.05.025 31100624

[53] Reshetnikov AN. Hygrotactic and olfactory orientation in juvenile common toads (Bufo bufo) during the postmetamorphic period. Adv Amphib Res Former Sov Union. 1996;1: 181–190.

[54] Farabaugh NF, Nowakowski AJ. Behavioral responses of the strawberry poison frog (Oophaga pumilio) to herbicide olfactory cues: Possible implications for habitat selection and movement in altered landscapes. Can J Zool. 2014;92: 979–984. 10.1139/cjz-2014-0111

[55] Swann JM, Schultz TW, Kennedy JR. The effects of the organophosphorous insecticides Dursban ^TM^ and Lorsban ^TM^ on the ciliated epithelium of the frog palate in vitro. Arch Environ Contam Toxicol. 1996;30: 188–194. 10.1007/BF00215797 8593081

[56] Dinehart SK, Smith LM, McMurry ST, Anderson TA, Smith PN, Haukos DA. Toxicity of a glufosinate- and several glyphosate-based herbicides to juvenile amphibians from the Southern High Plains, USA. Sci Total Environ. 2009;407: 1065–1071. 10.1016/j.scitotenv.2008.10.010 19000631

[57] Van Meter RJ, Glinski DA, Henderson WM, Purucker ST. Soil organic matter content effects on dermal pesticide bioconcentration in American toads (Bufo americanus). Environ Toxicol Chem. 2016;35: 2734–2741. 10.1002/etc.3439 27028289

[58] Shuman-Goodier ME, Propper CR. A meta-analysis synthesizing the effects of pesticides on swim speed and activity of aquatic vertebrates. Sci Total Environ. 2016;565: 758–766. 10.1016/j.scitotenv.2016.04.205 27261557

[59] Ockleford C, Adriaanse P, Berny P, Brock T, Duquesne S, Grilli S, et al Scientific Opinion on the state of the science on pesticide risk assessment for amphibians and reptiles. EFSA J. 2018;16 10.2903/j.efsa.2018.5125 32625798 PMC7009658

[60] Brühl CA, Pieper S, Weber B. Amphibians at risk? Susceptibility of terrestrial amphibian life stages to pesticides. Environ Toxicol Chem. 2011;30: 2465–2472. 10.1002/etc.650 21898550

[61] DuRant SE, Hopkins WA, Talent LG. Impaired terrestrial and arboreal locomotor performance in the western fence lizard (Sceloporus occidentalis) after exposure to an AChE-inhibiting pesticide. Environ Pollut. 2007;149: 18–24. 10.1016/j.envpol.2006.12.025 17360091

[62] Ingram EM, Augustin J, Ellis MD, Siegfried BD. Evaluating sub-lethal effects of orchard-applied pyrethroids using video-tracking software to quantify honey bee behaviors. Chemosphere. 2015;135: 272–277. 10.1016/j.chemosphere.2015.04.022 25966045

[63] Cordeiro EMG, Corrêa AS, Venzon M, Guedes RNC. Insecticide survival and behavioral avoidance in the lacewings Chrysoperla externa and Ceraeochrysa cubana. Chemosphere. 2010;81: 1352–1357. 10.1016/j.chemosphere.2010.08.021 20817256

[64] Aitbali Y, Ba-M’hamed S, Elhidar N, Nafis A, Soraa N, Bennis M. Glyphosate based- herbicide exposure affects gut microbiota, anxiety and depression-like behaviors in mice. Neurotoxicol Teratol. 2018;67: 44–49. 10.1016/j.ntt.2018.04.002 29635013

[65] Denoël M, Libon S, Kestemont P, Brasseur C, Focant JF, De Pauw E. Effects of a sublethal pesticide exposure on locomotor behavior: A video-tracking analysis in larval amphibians. Chemosphere. 2013;90: 945–951. 10.1016/j.chemosphere.2012.06.037 22824732

[66] Lajmanovich RC, Peltzer PM, Martinuzzi CS, Attademo AM, Bassó A, Colussi CL. Insecticide pyriproxyfen (Dragón®) damage biotransformation, thyroid hormones, heart rate, and swimming performance of Odontophrynus americanus tadpoles. Chemosphere. 2019;220: 714–722. 10.1016/j.chemosphere.2018.12.181 30611069

[67] Franco-Restrepo JE, Forero DA, Vargas RA. A Review of Freely Available, Open-Source Software for the Automated Analysis of the Behavior of Adult Zebrafish. Zebrafish. 2019;16: zeb.2018.1662. 10.1089/zeb.2018.1662 30625048

[68] Allentoft M, O’Brien J. Global amphibian declines, loss of genetic diversity and fitness: a review. Diversity. 2010;2: 47–71. 10.3390/d2010047

[69] Brühl CA, Zaller JG. Biodiversity Decline as a Consequence of an Inappropriate Environmental Risk Assessment of Pesticides. Front Environ Sci. 2019;7: 2013–2016. 10.3389/fenvs.2019.00177

