# Supplementary material for: Avoidance behavior of juvenile common toads (Bufo bufo) in response to surface contamination by different pesticides
Source: PLoS One. 2020 Nov 30;15(11):e0242720. doi: 10.1371/journal.pone.0242720 (PMC7704001; doi:10.1371/journal.pone.0242720)
Supplement: S1 Table — The table includes the date when the toads were captured, the date of the experimental run, the times when the first and the last toad were placed in the test arenas, the mean weight with its standard deviation (SD) of the toads used in each test as well the proportion of the total time a toad spent in a 2.5 cm wide area at the border between the contaminated and uncontaminated side of an area (buffer zone). Positions of toads in the buffer zone were excluded when analyzing the avoidance behavior. FRmax is the maximum recommended field rate of a formulation. (DOCX) [file pone.0242720.s001.docx]

**S1 Table. Detailed information about each choice test.** The table includes the date when the toads were captured, the date of the experimental run, the times when the first and the last toad were placed in the test arenas, the mean weight with its standard deviation (SD) of the toads used in each test as well the proportion of the total time a toad spent in a 2.5 cm wide area at the border between the contaminated and uncontaminated side of an area (buffer zone). Positions of toads in the buffer zone were excluded when analyzing the avoidance behavior. FR_max_ is the maximum recommended field rate of a formulation.

| **Formulation** | **% of FR_max_** | **Date of toad sampling** | **Date of test** | **Start time first toad** | **Start time last toad** | **Weight (mg)** | | **Time in buffer zone (%)** | | | |
| --- | --- | --- | --- | --- | --- | --- | --- | --- | --- | --- | --- |
|  |  |  |  |  |  | **Mean** | **SD** | **Median** | **IQR** | **Range** | **Mean** |
| Folpan® 500 SC | 100 | 01.08.2018 | 14.08.2018 | 12:17 | 12:34 | 293.1 | 42.6 | 6.1 | 2.7 - 15.9 | 1.5 - 46.6 | 12.7 |
| Folpan® 500 SC | 10 | 24.08.2018 | 30.08.2018 | 11:16 | 11:33 | 372.7 | 48.7 | 5.8 | 4.3 - 11.5 | 0.8 - 52.9 | 12.5 |
| Folpan® 80 WDG | 100 | 03.09.2018 | 13.09.2018 | 11:14 | 11:30 | 433.7 | 115.5 | 12.2 | 6.4 - 24.8 | 2.0 - 43.3 | 17.1 |
| Folpan® 80 WDG | 10 | 01.08.2018 | 07.08.2018 | 12:27 | 12:42 | 368.6 | 52.3 | 15.8 | 10.2 - 34.6 | 2.0 - 45.2 | 20.0 |
| Funguran® progress | 100 | 09.08.2018 | 16.08.2018 | 12:26 | 12:44 | 306.6 | 49.8 | 10.2 | 4.5 - 16.2 | 3.2 - 36.2 | 12.2 |
| SpinTor^TM^ | 100 | 29.08.2018 | 11.09.2018 | 11:45 | 12:04 | 442.1 | 84.9 | 13.9 | 6.1 - 21.2 | 1.5 - 40.4 | 15.1 |
| Taifun® forte | 100 | 25.07.2018 | 09.08.2018 | 13:29 | 13:45 | 333.8 | 71.7 | 10.5 | 6.7 - 15.4 | 3.8 - 60.2 | 15.0 |
| Taifun® forte | 10 | 13.08.2018 | 28.08.2018 | 10:58 | 11:20 | 362.4 | 58.6 | 22.4 | 11.0 - 31.6 | 5.3 - 59.3 | 24.2 |
| Vivando® | 100 | 09.08.2018 | 21.08.2018 | 12:27 | 12:43 | 305.5 | 60.8 | 8.2 | 3.0 - 17.5 | 1.6 - 54.9 | 14.9 |
| Vivando® | 10 | 24.08.2018 | 04.09.2018 | 11:07 | 11:20 | 384.6 | 75.0 | 12.1 | 4.8 - 35.5 | 2.1 - 58.5 | 20.6 |
| Wettable Sulphur Stulln | 100 | 19.09.2018 | 20.09.2018 | 12:07 | 12:24 | 552.6 | 88.6 | 7.8 | 3.9 - 18.6 | 2.7 - 51.6 | 13.7 |
