# Supplementary figures and images for: Avoidance behavior of juvenile common toads (Bufo bufo) in response to surface contamination by different pesticides
Source: PLoS One. 2020 Nov 30;15(11):e0242720. doi: 10.1371/journal.pone.0242720 (PMC7704001; doi:10.1371/journal.pone.0242720)

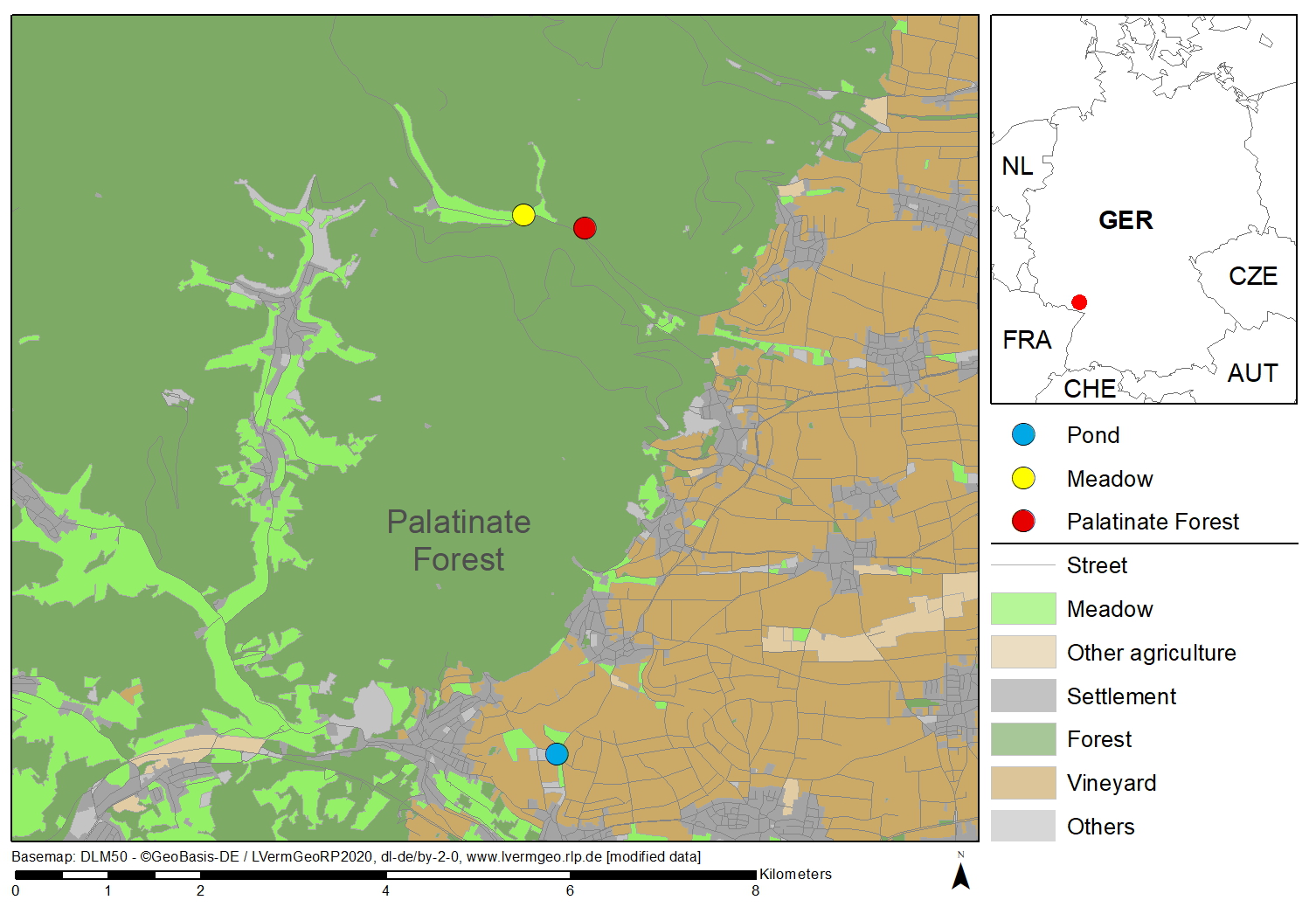

Supplement: S1 Fig — The points show the location of the pond where the individuals for the experimental runs were captured (blue; "Pond"), the location where insects for feeding of the toads were captured (yellow; "Meadow") and the location where soil, moss and leaves were collected to equip the outdoor net cages (red; "Palatinate Forest"). Reprinted from www.lvermgeo.rlp.de under a CC BY license, with permission from GeoBasis-DE / LVermGeoRP2020, original copyright 2020. (JPG) [file pone.0242720.s002.jpg]

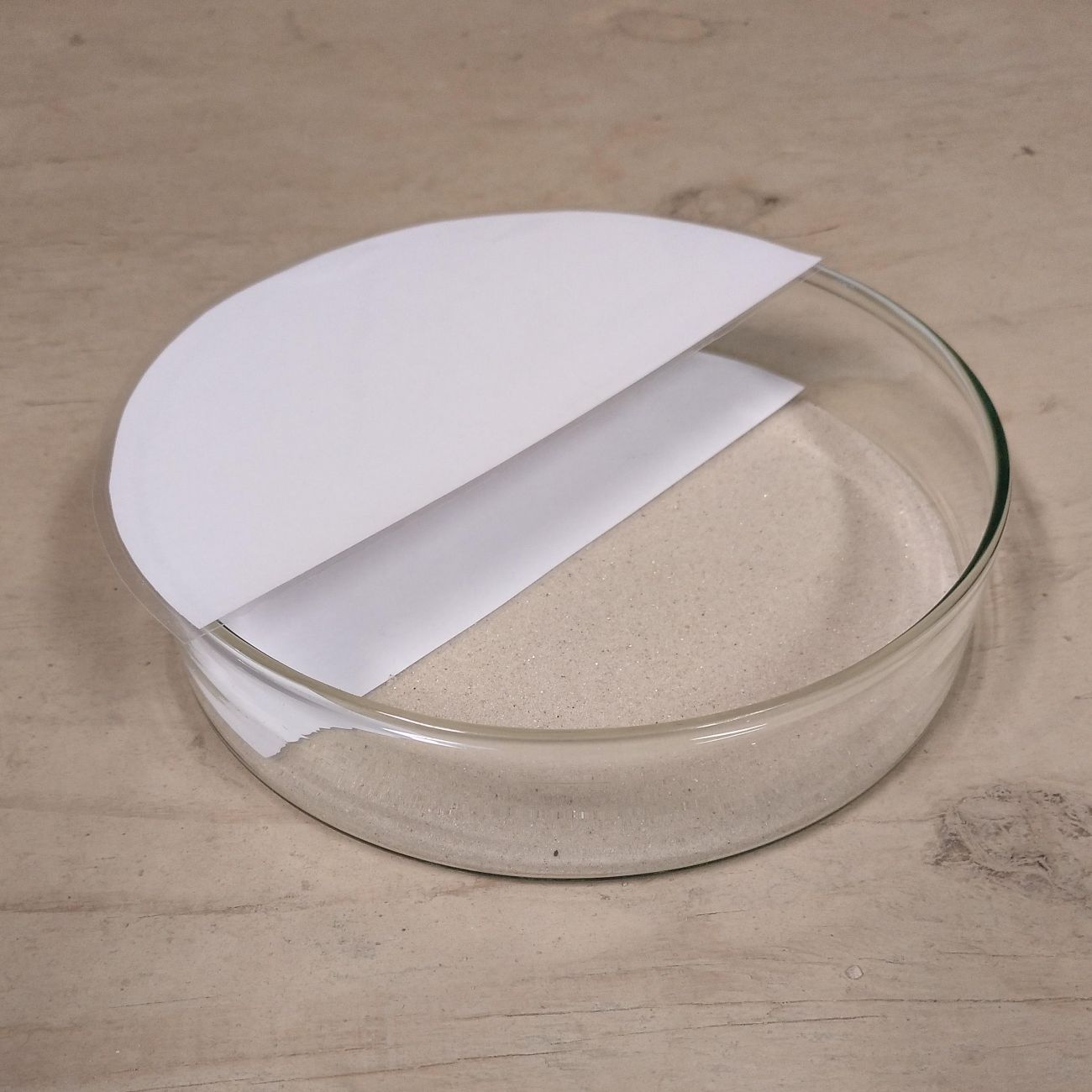

Supplement: S2 Fig — One side is covered with laminated paper semicircles to prevent contamination of the clean side during the application process. (JPG) [file pone.0242720.s003.jpg]

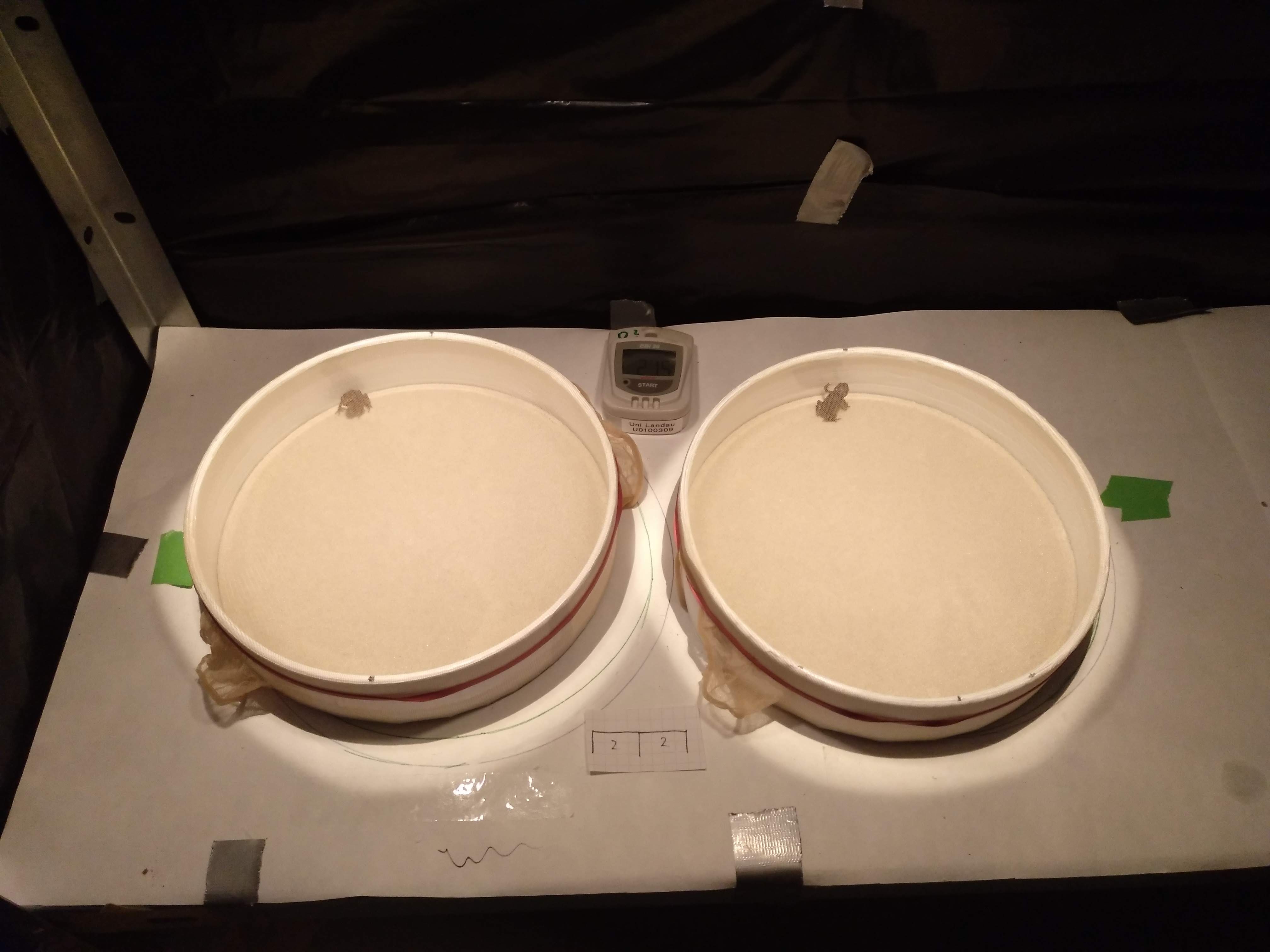

Supplement: S3 Fig — Arenas are covered with a polyamide fabric. (JPG) [file pone.0242720.s004.jpg]

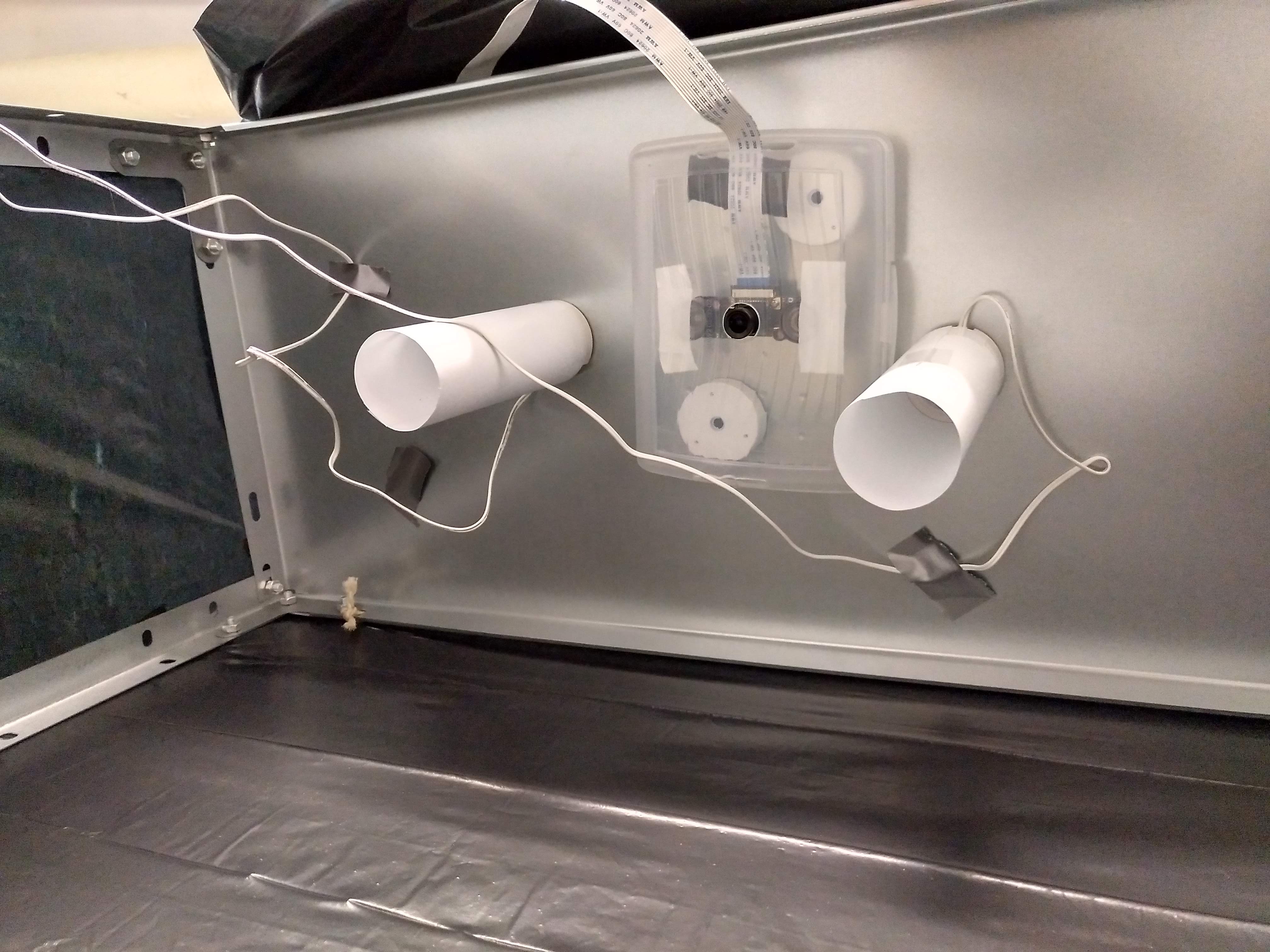

Supplement: S4 Fig — The camera was attached to a Raspberry Pi (Raspberry Pi 3 Model B; Raspberry Pi Foundation, Cambridge, UK). (JPG) [file pone.0242720.s005.jpg]

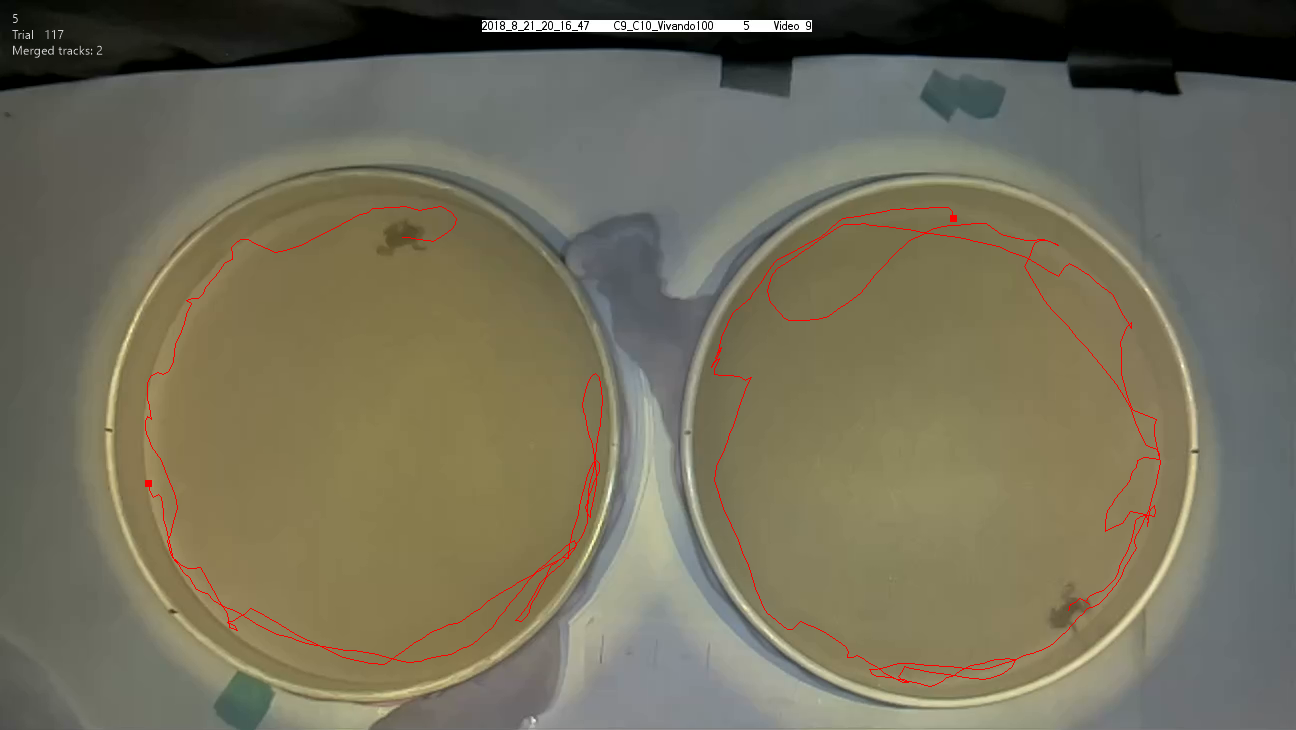

Supplement: S5 Fig — (PNG) [file pone.0242720.s006.png]
